# Sequence comparisons of plasmids pBJS-O of *Spiroplasma citri *and pSKU146 of *S. kunkelii*: implications for plasmid evolution

**DOI:** 10.1186/1471-2164-6-175

**Published:** 2005-12-07

**Authors:** Bharat D Joshi, Michael Berg, Janet Rogers, Jacqueline Fletcher, Ulrich Melcher

**Affiliations:** 1 Department of Biochemistry and Molecular Biology, Oklahoma State University, 246 NRC, Stillwater, OK 74078, USA; 2 Department of Entomology and Plant Pathology, Oklahoma State University, 127 NRC, Stillwater, OK 74078, USA; 3 P&K Microbiology Services, Inc. 1936 Olney Ave., Cherry Hill, NJ 08003, USA

## Abstract

**Background:**

*Spiroplasma citri *BR3-3X and *S. kunkelii *CR2-3X cause serious diseases worldwide on citrus and maize species, respectively. *S. citri *BR3-3X harbors a plasmid, pBJS-Original (pBJS-O), that encodes the spiroplasma adhesion related protein 1 (SARP1), a protein implicated in binding of the pathogen to cells of its leafhopper vector, *Circulifer tenellus*. The *S. kunkelii *CR2-3X plasmid, pSKU146, encodes a homolog of SARP1, Sk-ARP1. Due to the close phylogenetic relationship of the two pathogens, we hypothesized that the two plasmids are closely related as well.

**Results:**

The nucleotide sequence of pBJS-O was determined and compared to the sequences of a plasmid from BR3-T (pBJS-T), which is a multiply passaged leafhopper transmissible derivative of BR3-3X, and to known plasmid sequences including that of pSKU146. In addition to *arp1*, the 13,374 bp pBJS-O sequence putatively contains nine genes, recognized as open reading frames (ORFs). Several pBJS-O ORFs have homologs on pSKU146. However, the sequences flanking *soj*-like genes on both plasmids were found to be more distant from one another than sequences in any other region. Further, unlike pSKU146, pBJS-O lacks the conserved *oriT *region characteristic of the IncP group of bacterial plasmids. We were unable to identify a region in pBJS-O resembling a known plasmid origin of transfer. In regions where sequence was available for the plasmid from both BR3-3X and BR3-T, the pBJS-T sequence had a 0.4 kb deletion relative to its progenitor, pBJS-O. Southern blot hybridization of extrachromosomal DNA from various *S. citri *strains and spiroplasma species to an *arp*-specific probe and a probe made from the entire plasmid DNA of BR3-3X revealed limited conservation of both sequences in the genus *Spiroplasma*. Finally, we also report the presence on the BR3-3X chromosome of *arp2*, an *S. citri *homolog of *arp1 *that encodes the predicted protein SARP2. The C-terminal domain of SARP2 is homologous to that of SARP1, but its N-terminal domain is distinct.

**Conclusion:**

Our data suggest that pBJS is a novel *S. citri *plasmid that does not belong to any known plasmid incompatibility group. The differences between pBJS-O and pSKU146 suggest that one or more events of recombination have contributed to the divergence of the plasmids of the two sister *Spiroplasma *species; the plasmid from *S. citri *itself has diverged slightly during the derivation of *S. citri *BR3-T from BR3-3X. Our data also show that pBJS-O encodes the putative adhesin SARP1. The presence of *traE *and *mob *on pBJS-O suggests a role for the plasmid in spiroplasmal conjugation.

## Background

The phytopathogenic spiroplasmas and phytoplasmas, which cause serious diseases of economically important plant species worldwide [[1] and [2]], are wall-less prokaryotes phylogenetically related to Gram-positive eubacteria with low G+C content [3]. They are transmitted in nature by phloem-feeding insects, predominantly leafhoppers, in a propagative manner [4]. Even though there are close to forty recognized spiroplasma species, only three plant pathogenic spiroplasmas have been identified and characterized to date. *S. citri *[[5,6] and [7]] is the causative agent of stubborn disease of citrus and brittle root disease of horseradish; *S. kunkelii *[[8,9] and [10]] is the etiological agent of corn stunt; and *S. phoeniceum *[11] causes periwinkle yellows. Unlike phytoplasmas, spiroplasmas can be cultured *in vitro*. Therefore, the relationships between *S. citri *and its insect vectors, the beet leafhopper, *Circulifer tenellus*, and the related species, *C. haematoceps *[12], have been investigated extensively, serving as models for investigating the molecular aspects of mollicute-vector interactions.

Spiroplasma binding to insect host and non-host cells, both in tissue-culture and within the intact insect, has been reported [[13] and [14]]. The loss and restoration of the ability of *S. citri *to adhere to tissue-cultured *C. tenellus *cells was associated with degradation and restoration of P89 (designated SARP1), a spiroplasma membrane protein [14]. Due to the possible direct involvement of SARP1 in the spiroplasma-leafhopper interaction, it was hypothesized that SARP1 is an adhesin. Later, Berg *et al *[15] reported cloning and characterization of *arp1*, the gene encoding SARP1, from *S. citri *BR3-T. They also reported that mature SARP1 protein contains a novel domain at the N-terminus, called "sarpin", made of six repeats of 39–42 amino acids each.

*S. citri *harbors several extrachromosomal DNAs with unique restriction patterns [[16-18] and [19]]. *S. citri *lines, derived from a clone, and sister clones of the same lines showed differences in their extrachromosomal DNAs [20]. In addition to known plasmids, there are replicative forms (RFs) of several viruses and other uncharacterized circular extrachromosomal DNAs in *S. citri *[21].

Plasmids have also been noted in strains of *S. kunkelii *[22]. Recently, Davis and colleagues [23] reported the complete sequence of the *S. kunkelii *CR2-3X plasmid pSKU146, which encodes a homolog of SARP1, Sk-ARP1. In the present study, we isolated and characterized a related indigenous plasmid, designated pBJS-Original (pBJS-O), from *S. citri *BR3-3X. This is a report of the discovery, distribution and characterization of that plasmid. Among other genes, pBJS-O contains *arp1*. The significance of the discovery of pBJS-O in relation to our current understanding of the *S. citri*-leafhopper interactions and potential genetic manipulations in mollicutes is discussed. Implications for the evolution of both pBJS-O and pSKU146 are also presented.

## Results

### Detection and analysis of *arp2*

SARP1 has been characterized previously and the gene encoding it, *arp1*, has been cloned and sequenced [GenBank:AJ297706] from *S. citri *BR3-T [15]. In the process, an *Rsa*I restriction fragment was cloned and sequenced from BR3-T genomic DNA; the alignment of this fragment with AJ297706 revealed 92% similarity in the 3' 660 nucleotides of the former sequence. However in the 5' 55 bases of the total 715 bp, upstream from position 2370 in AJ297706, the new fragment was not similar to the known sequence. We designated this gene, which resembles but is not identical to *arp1*, as *arp2 *and its putative protein product as SARP2. As also noted by Bai *et al*. [22], the *S. kunkelii *CR2-3X genome contains two sequences similar to those of *S. citri *BR3-T *arp *genes. The predicted protein, Sk-ARP1 (for *S. kunkelii *adhesion related protein 1), encoded by the first sequence, *Sk-arp1*, contains seven rather than six sarpin repeats and has C-terminal domains resembling those of SARP1 [15]. The second sequence encodes a putative protein whose C-terminus is homologous to that of SARP1, but has an unrelated N-terminus. This protein is designated Sk-ARP2 (*S. kunkelii *adhesion related protein 2) and the corresponding gene is named *Sk*-*arp2*. SARP1 has sequence similarity with known adhesins. Fleury *et al*. [24] have shown that the predicted amino acid sequence of P40, a *Mycoplasma agalactiae *cytadhesin, is similar not only to that of SARP1 but also to the one of P50, an adhesin of *M. hominis*.

### Isolation and distribution of *Spiroplasma *extrachromosomal DNA

**Figure 1:**
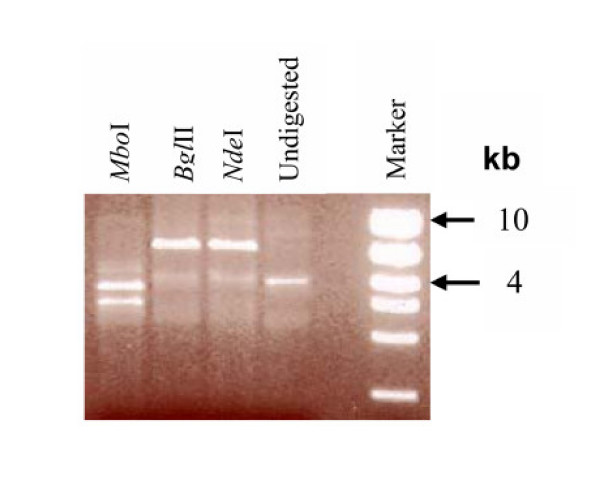
Restriction digests of pBJS-O DNA with *Mbo*I, *Bgl*II and *Nde*I. The marker used was the High Mass Ladder (Invitrogen Corp., Carlsbad, CA, USA). Sizes of the fragments are denoted in kb.

We isolated extrachromosomal DNA from *S. citri *BR3-3X to test the hypothesis that this DNA contains an *arp*-like gene as in *S. kunkelii*. Restriction of the DNA with single enzymes, including *Bgl*II and *Nde*I, converted a DNA migrating with 9 kb into a fragment migrating close to 7 kb (Fig. 1). These results were consistent with the presence of a single major plasmid. We designated the plasmid pBJS-O. By nucleotide sequencing, we determined that the actual size of the plasmid was 13,374 bp and deposited the sequence in the EMBL Nucleotide Sequence Database [EMBL:AJ972409]

**Figure 2:**
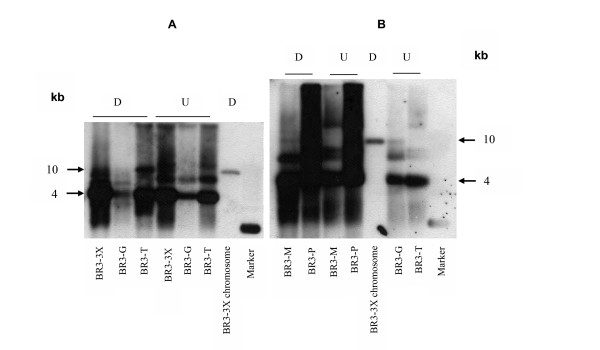
Southern blotting hybridization of (A) *S. citri *BR3-3X, BR3-G, BR3-T, and (B) *S. citri *BR3-M and BR3-P plasmid preparations to an *arp1*-derived probe. *Eco*RI-digested *S. citri *BR3-3X chromosomal DNA and *Eco*RI-digested and undigested plasmid preparations from BR3-3X, BR3-G, BR3-T, BR3-M and BR3-P are shown. D, digested with *Eco*RI; U, undigested. Hybridization in the marker lane is due to presence of short pBluescript vector sequences in the probe.

**Figure 3:**
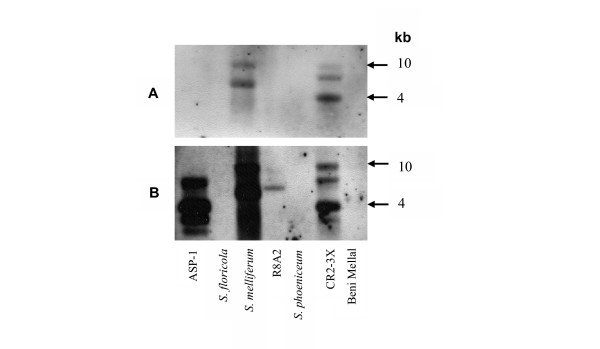
Southern blotting hybridization of undigested plasmid preparations from different *S. citri *strains and spiroplasma species to (A) an *arp1*-derived probe and (B) the whole pBJS-O probe. Plasmids from *S. citri *ASP-1, R8A2 and Beni Mellal, and from *S. floricola*, *S. melliferum*, *S. phoeniceum *and *S. kunkelii *CR2-3X were used. The blot shown in panel A was stripped and rehybridized using the whole pBJS-O probe, shown in panel B.

To test the conservation of pBJS-O in *S. citri *strains derived from *S. citri *BR3, plasmid preparations from *S. citri *BR3-3X and from BR3-G, BR3-T, BR3-M and BR3-P, lines derived from BR3-3X, were probed with a DNA fragment derived from *arp1 *(Fig. 2 and Table 1). All hybridized with the probe, producing two or more bands. To test the conservation of pBJS-O in other *S. citri *strains, other plant-associated spiroplasmas and the closest relative of *S. citri*, *S. melliferum *[25], the plasmids of *S. kunkelii *CR2-3X, *S. melliferum*, *S. citri *strains R8A2, ASP-1 and Beni Mellal, *S. floricola *and *S. phoeniceum *also were probed with the *arp1*-derived probe (Fig. 3A). Only *S. kunkelii *CR2-3X and *S. melliferum *reacted in the hybridization. However, when the same plasmids were probed with the whole pBJS-O plasmid as a probe (Fig. 3B), all the sample preparations, except those from *S. citri *Beni Mellal, *S. floricola *and *S. phoeniceum*, hybridized with the probe. All of the above Southern hybridization experiments revealed multiple reactive species in the plasmid preparations and the hybridization patterns of *Eco*RI-digested and undigested plasmid samples were very similar to each other. For comparison, the blots included *Eco*RI-digested chromosomal DNA of *S. citri *BR3-3X. A single hybridization signal distinct from those of plasmid preparations was observed (Fig. 2).

**Table 1:** Results of the Southern hybridizations of undigested plasmid preparations from various spiroplasma species and *S. citri *strains to either an *arp1*-derived probe or whole pBJS-O probe. + and - denote positive and negative hybridizations, respectively.

**Spiroplasma**		**Probe**		**Biological features**	
Species*	Strain	*arp1*	pBJS-O	Transmissibility	Pathogenicity

	BR3-3X	+	+	+	+
	BR3-G	+	+	-	-
	BR3-T	+	+	+	+
*S. citri*	BR3-M	+	+	+	+
	BR3-P	+	+	Very low	-
	ASP-1	-	+	Unknown	Unknown
	R8A2	-	+	Unknown	Unknown
	Beni Mellal	-	-	Unknown	Unknown
*S. kunkelii*	CR2-3X	+	+	+	+
*S. phoeniceum*	P40	-	-	+**	+
*S. melliferum*	TS2	+	+	Unknown	-
*S. floricola*	23-6	-	-	Unknown	-

*S. citri, *S. kunkelii*, *S. phoeniceum *and *S. melliferum *belong to serogroup I, whereas *S. floricola *belongs to serogroup III. The biological features of the spiroplasmas, except for *S. phoeniceum*, are taken from references 25 and 46.

**Only experimental transmission to the plant host is known for this spiroplasma. It is unclear whether it can be naturally transmitted by leafhoppers.

### *arp1 *and *arp2 *locations in *S. citri *BR3-3X

**Figure 4:**
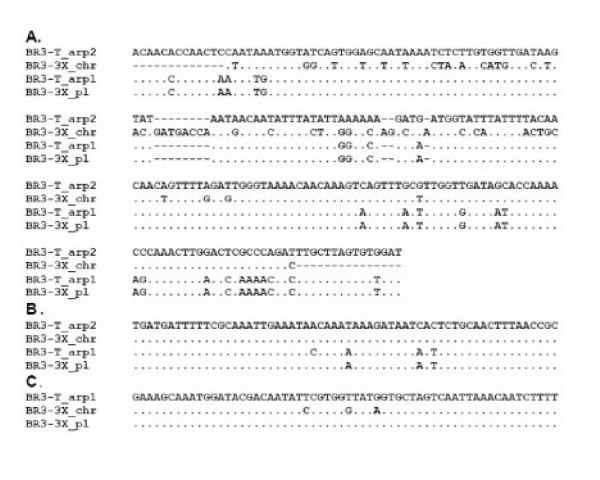
Comparison of partial nucleotide sequences of *S. citri *BR3-3X chromosomal (BR3-3X_chr) and plasmid (BR3-3X_pl) sequences to those of available BR3-T *arp1 *and *arp2 *genes (BR3-T_arp1 and BR3-T_arp2, respectively). Only the regions containing polymorphic positions are shown. (A) region of *arp1 *positions 3572 to 3800 (AJ297706). (B) region of *arp1 *positions 4118 to 4177 and (C) region of *arp1 *positions 4658 to 4717). *S. citri *BR3-T *arp2 *is not available for the last sequence alignment. Gaps are denoted by dashed lines, whereas dots denote identical bases.

**Figure 5:**
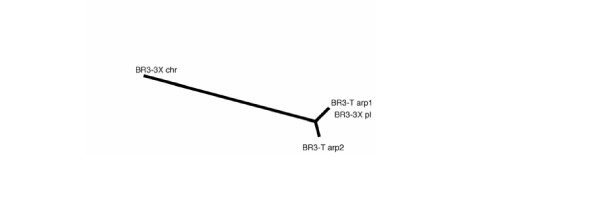
An unrooted phylogenetic tree representing the nucleotide sequence alignment shown in Fig. 4A. The tree was generated using algorithms ClustalW and PHYLIP from the Biology Workbench using the neighbour-joining method.

The Southern blot hybridization results suggest that *arp*-related sequences are present on both a plasmid and the chromosome. *arp1 *and *arp2 *from BR3-T are nearly identical over a considerable portion of their nucleotide sequence. Hence, using a probe containing this conserved region should detect both genes. Nevertheless, *arp1 *and *arp2 *differ at several positions in those regions. To determine whether the BR3-3X plasmid and chromosomal sequences represented *arp1 *or *arp2 *genes, we determined parts of the sequences of BR3-3X plasmid and chromosomal DNAs by direct sequencing and by sequencing amplified PCR products. Comparison of the BR3-3X *arp *sequences with those of BR3-T revealed that the BR3-3X *arp2 *sequence had diverged more from the other three sequences than the latter had from each other (Figs. 4 and 5). At positions where the two BR3-T genes differed from one another, the chromosomal BR3-3X sequence had *arp2 *residues in 21 positions and *arp1 *residues in only 3 positions (Figs. 4A and 4B). Conversely, at *arp1*- and *arp2*-specific positions, the BR3-3X plasmid DNA had no *arp2 *residues and 28 *arp1 *residues. Further, at all 57 positions at which chromosomal and plasmid sequences differed, the BR3-3X plasmid and *arp1 *nucleotides were identical. Hence, we conclude that, in *S. citri *BR3-3X, the *arp1 *gene resides on a plasmid and that the *arp2 *gene most likely resides on the chromosome. The newly determined *arp2 *sequences from BR3-3X and BR3-T were deposited in the EMBL Nucleotide Sequence Database [EMBL:AM040506 and EMBL:AM040505, respectively].

### Complete pBJS-O sequencing and analysis

The 4273 bp sequence [GenBank:AJ297706] originally cloned and characterized from *S. citri *BR3-T [15] contains a partial ORF *soj*, followed by ORF2, P89 (*arp1*) and another partial ORF, ORF4. AJ297706 was used to design primers and initiate primer walking to determine the complete pBJS-O plasmid sequence and allow its characterization. During sequencing, a segment (from nucleotide 1–80) of the assembled sequence proved particularly difficult to sequence. It contained three of the six oligopurine/oligopyrimidine tracts of 12 or more bp in the entire plasmid sequence. That the sequence of the tracts was consistent with triple-helix formation suggests that this region of the plasmid may readily form triple-helical structures interfering with sequencing.

**Figure 6:**
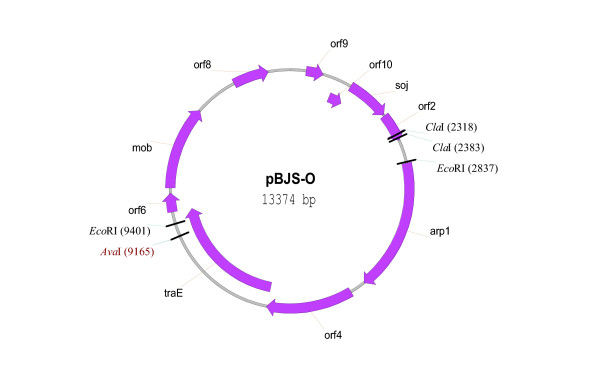
The ORF and restriction map of pBJS-O.

The total plasmid sequence is 13,374 bp in length and contains ten predicted ORFs (Fig. 6 and Table 2), of which *orf2 *(*S. citri *ORF2) has no homologs, and *orf9 *and *orf10 *appear to have distant relatives (E values 0.34 and 0.024, respectively). Of the ten, six putative pBJS-O-ORFs have homologs in pSKU146, the recently characterized *S. kunkelii *CR2-3X plasmid [16]: *arp1 *(adhesin protein; E value 0.0), *orf4 *(hypothetical protein pSKU146_11; E value 0.0), *traE *(conjugation ATPase; E value 0.0), *orf6 *(hypothetical protein pSKU146_13; E value 9 × 10^-45^), *mob *(mobilization protein; E value 0.0) and *orf8 *(hypothetical protein pSKU146_17; E value 1 × 10^-103^). Predicted products of *traE *and *mob *are similar to proteins involved in conjugative DNA transfer in other bacterial genera. In the regions where the plasmid sequence was available from both BR3-3X and BR3-T, pBJS-T (the plasmid from *S. citri *BR3-T) sequence had a 0.4 kb deletion relative to pBJS-O, bringing the *orf4 *gene close to *arp1 *and *traE*. In BR3-3X, however, *arp1 *and *orf4 *are separated by 281 bp. The nucleotide sequence variations between pBJS-O and pBJS-T were found to be clustered. Two regions of enhanced variation were observed over a 200 bp stretch in the ORF2-*arp1 *intergenic region (positions 2700 to 2900 in pBJS-O). In a comparable stretch from position 5262 to 5544 in the *arp1*-ORF4 intergenic region, a single stretch of dissimilarity was found.

**Table 2:** Descriptions of ORFs present on pBJS-O.

**ORF #**	**Map Position**	**Length (bp)**	**Closest homolog (from BLASTP search)**	**E value**
1	1114–1896	783	Soj-like protein [*S. citri*]*	1 × 10^-116^
2	1916–2434	519	hypothetical protein [*S. citri*]*	1 × 10^-100^
3	2859–5255	2397	putative adhesin P89 [*S. citri*]*	0
4	5536–7101	1566	hypothetical protein [*S. kunkelii*]	0
5	7091–9613	2523	conjugation ATPase [*S. kunkelii*]	0
6	9617–9955	339	hypothetical protein [*S. kunkelii*]	9 × 10^-45^
7	10047–11558	1512	mobilization protein [*S. kunkelii*]	0
8	12338–12988	651	hypothetical protein [*S. kunkelii*]	1 × 10^-103^
9	270–581	312	Unknown	0.34
10	831–1127	297	Unknown	0.024

* AJ297706, the sequence originally characterized from *S. citri *BR3-T, is the source of these hits.

**Figure 7:**
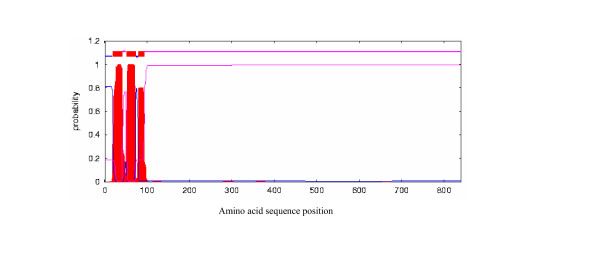
Predicted locations of transmembrane helices and intervening loops in the putative protein encoded by ORF5 (*traE*) of pBJS-O. The sequential amino acid positions in the primary sequence of the polypeptide are on the X-axis, while the probability score of each residue for being in a transmembrane helix is on the Y-axis in red. The blue and pink curves denote the probability of each amino acid in the sequence to be cytosolic or extracellular, respectively. In the schematic representation of the protein domains at the top, blue lines show the cytosolic portions, purple ones denote the extracellular portions and the thick horizontal bars denote the predicted transmembrane portions of the polypeptide, respectively.

Algorithm TMHMM v. 2.0 was used to predict the locations of transmembrane helices and intervening loops in the putative products of *traE *(Fig. 7), *mob *and *orf4*. Although the TraE polypeptide was predicted to contain three transmembrane helices, the third helix was predicted at a lower probability than were the other two. Assuming the presence of three transmembrane helices, the protein was predicted to have the N-terminal region (about 10% of the length of the polypeptide) in the cytosol and almost all of the rest of the protein extracellular.

Plasmid pSKU146 from *S. kunkelii *CR2-3X encodes the *S. kunkelii *homolog of SARP1, Sk-ARP1. In addition to *sk*-*arp1*, pSKU146 contains 17 ORFs. The pSKU146-ORFs having counterparts on pBJS-O were listed above. However, although both plasmids contain genes encoding the ParA-like protein, Soj, sequences surrounding those genes are more distant from one another than are sequences in any other regions. Further, unlike pSKU146, pBJS-O lacks the conserved *oriT *region characteristic of the IncP group of bacterial plasmids. Also, we were unable to identify a region in pBJS-O resembling a known plasmid origin of transfer.

## Discussion

In the present study we report isolation, distribution and structural characterization of pBJS-O, an indigenous *S. citri *BR3-3X plasmid. We also present evidence that pBJS-O harbors *arp1*, the gene encoding SARP1, and describe the presence on the BR3-3X chromosome of *arp2*, an *S. citri *homolog of *arp1*. Finally, the sequences of pBJS-O, pBJS-T and the *S. kunkelii *CR2-3X plasmid, pSKU146, in relation to plasmid evolution are discussed.

### Conservation of *arp *and pBJS-O sequences in *Spiroplasma*

In Southern hybridizations, the similarity in the hybridization patterns of *Eco*RI digested versus undigested pBJS-O preparations, despite the presence of two GAATTC recognition sequences, may be due to an adenine methylation system in *S. citri*. Restriction site modification in *S. citri *has been reported elsewhere. Rascoe *et al*. [26] detected multiple bands of *S. citri *extrachromosomal DNA by Southern blotting, which they attributed to incomplete restriction due to variable restriction site modification in the DNA, and Ye *et al*. [27] reported protection of an *Eco*RI site in the *S. citri *16S rDNA. Moreover, differential methylation of restriction sites in the RF of the spiroplasma virus, SVTS2, allowed Sha *et al*. [28] to clone the full-length DNA.

*S. citri *BR3-3X showed probe-reactive sequences in both the chromosomal and extrachromosomal DNA fractions. However, that the patterns of hybridization of the two fractions differed significantly from each other demonstrates that the two fractions of BR3-3X DNA were not appreciably cross-contaminated. Sequence analyses of DNA from the two fractions showed that, in BR3-3X, *arp1 *resides on pBJS-O and *arp2 *on the chromosome. Hybridization of *S. citri *ASP-1 and R8A2 plasmid preparations with the pBJS-O probe (Fig. 3B), but not with the *arp1 *probe (Fig. 3A), indicates that each of these two strains contained a plasmid related to pBJS-O, which differed from pBJS-O in lacking *arp1*. Although *S. citri *ASP-1 and R8A2 were originally derived from the same parent strain, both have undergone extensive cultivation *in vitro *since their first isolation, which may have contributed to the differences between their plasmids and pBJS-O. Moreover, the differences in the maintenance regimes of the various spiroplasmas tested may have contributed to the evolution of their plasmids. In this paper we could not correlate pBJS-O and pBJS-O like sequences with either transmissibility or phytopathogenicity of the spiroplasmas tested. However, it is still hypothesized that SARP1 is involved in *S. citri *transmission by the insect vector.

Frequent chromosomal rearrangements such as inversions and deletions, leading to genome instability, have been reported in spiroplasmas, such as in the lines derived from *S. citri *strain BR3 [[29] and [30]]. In the present study, we detected a 0.4 kbp deletion in pBJS-T relative to pBJS-O. Unlike BR3-3X, which was stored frozen, *S. citri *BR3-T was maintained for several years in turnip plants via transmission by the natural insect vector *C. tenellus*, possibly leading to the sequence differences between pBJS-T and pBJS-O. A recombinational chromosomal rearrangement is indicated by the 5'-sequence differences between *arp1 *and *arp2 *reported above.

Recombination likely also played a role in the generation of pBJS-O like plasmids. The gene organization on pBJS-O is similar to that of the recently characterized IncP-like *S. kunkelii *CR2-3X plasmid, pSKU146. Yet, the two plasmids have substantially different sequences in the region including the *soj*-like gene in both plasmids and the IncP *oriT *sequence in pSKU146. Highly similar sequences in the remainder of the two plasmids suggest that recombination events have occurred during the generation of one or both plasmids.

Phage sequences have been implicated in many recombination events in spiroplasmas. Only a short region with similarity to a phage gene was found in pBJS-O. However, the observation of strong stops to sequencing reactions in the region of nucleotides 1 to 80 is reminiscent of a strong stop encountered during the sequencing of the SVTS2 phage [31]. This strong stop was attributed to potential secondary structure putatively involved in phage packaging. It is, thus, possible that pBJS-O has some phage-like properties.

### pBJS-O genes

As mentioned above, ORF3, encoding SARP1, and adjacent ORFs [GenBank:AJ297706], had been cloned and characterized from *S. citri *BR3-T [15]. ORF3 was flanked downstream by a partial ORF (ORF4) having no known homologs. Upstream, ORF3 was flanked by ORF2, encoding a hypothetical protein with no similarity to any known protein, and ORF1, a partial ORF encoding a putative homolog of a ParA-like protein, Soj, which oscillates from pole to pole [32] and is important for chromosome partitioning in *Bacillus subtilis *[33]. In this study, the putative protein product of *orf4 *was predicted to contain eight transmembrane helices. Due to a 0.4 kb deletion in the derivation of pBJS-T, *orf4 *is possibly a part of the same transcription unit as *arp1 *and *traE *in this strain. In BR3-3X, *arp1 *and *orf4 *are separated by 281 bp, suggesting that they are transcribed separately. Consistent with different translational constraints on this region in BR3-T and BR3-3X, this region contains a large proportion of the differences between the lines. The translation start site of *traE *was predicted to be ten nucleotides upstream of the *orf4 *translation stop site.

Consistent with the observation of Bai *et al*. [22], putative products of the other pBJS-O ORFs, *traE *[34] and *mob *[35], are homologous to proteins that are components of the bacterial type IV secretion system involved in conjugative DNA transfer. Members of the TraE family of proteins are thought to form pili that, in addition to conjugation, are involved in processes like virus infection and biofilm formation. Bai *et al*. [22] reported the presence of three conserved transmembrane helices in four TraE homologs that they characterized from *S. kunkelii *M2. Ozbek *et al*. [36], in their transmission electron micrographs, reported the presence of structures resembling fimbriae and pili in *S. kunkelii *and Bai *et al*. [22] considered whether the structures may be involved in conjugation. Bové [37] reported that rod-shaped spiroplasma viruses, approximately 230–280 by 10–15 μm in size, can also be surface-associated. Because they can attach perpendicularly to the host membrane at their tips [[38] and [39]], they might resemble the structures reported as pili/fimbriae. In the putative TraE homolog reported here, unlike its *S. kunkelii *counterpart, two transmembrane helices were predicted at high probability and a third one at moderate probability. Should the third not actually be a transmembrane helix, the ATP binding site would be located intracellularly rather than extracellularly.

### pBJS-O gene organization and evolution

Unlike pSKU146, pBJS-O was found to lack the conserved *oriT *region characteristic of the IncP group of plasmids. We were also unable to identify a region in pBJS-O resembling any other known plasmid origins of transfer, suggesting that pBJS-O belongs to a hitherto unidentified group of plasmids. Horizontal transfer of a promiscuous plasmid, such as an IncP plasmid, between phylogenetically related and unrelated bacteria would help the hosts quickly adapt to different niches [40]. It is possible that an IncP-like plasmid was acquired by the ancestor of *S. citri *and *S. kunkelii*. The plasmid may have co-evolved with the host chromosomes after the divergence of the two species, leading to the emergence of pBJS-O and pSKU146, respectively, and to the adaptation of the pathogens to phylogenetically distinct leafhopper vectors and plant hosts.

### Future directions

Molecular genetic tools such as cloning and transposon-mediated mutagenesis are available for the study of mollicutes [41]. Cloned genes were expressed in *S. citri *GII-3 using artificial plasmids based on the *S. citri *chromosomal *oriC *[[42,43] and [44]]. However, those plasmids tend to integrate into the *S. citri *chromosome. When pCJ32, a derivative of the *oriC *plasmid pBOT1, containing an internal fragment of the gene *scm1 *(a motility-related *S. citri *gene), was transformed into *S. citri *GII-3 cells it successfully integrated into the host chromosome by homologous recombination and disrupted *scm1*, resulting in non-motile *S. citri *GII-3 mutants [45]. However, attempts to use pBOT1 in *S. citri *BR3-3X have been unsuccessful (F. Ye, *unpublished data*), possibly due to the incompatibility of the plasmid with the host. The indigenous *S. citri *BR-3X plasmid, pBJS-O, will help us develop a better vector for genetic manipulation not only in *S. citri *BR3-3X but also in other spiroplasmas.

## Conclusion

We have shown that the *S. citri *BR3-3X plasmid, pBJS-O, encodes the putative adhesin SARP1. This is the first report of an *S. citri *plasmid encoding a putative adhesin. We have further shown that the *arp1*-like gene, *arp2*, resides on the BR3-3X chromosome. The indigenous *S. citri *BR3-3X plasmid, pBJS-O, will be useful for the development of a better vector for genetic manipulation not only in *S. citri *BR3-3X but also in other spiroplasmas. Our data also suggest that pBJS-O is a novel *S. citri *plasmid that does not belong to any known plasmid incompatibility group. The differences between pBJS-O and pSKU146 suggest that recombination has contributed to the divergence of the two plasmids.

## Methods

### Spiroplasmas

*S. citri *BR3 was isolated from horseradish plants with brittle root disease [7]. *S. citri *BR3-T, derived from the triply cloned parental isolate (BR3-3X) by repeated transmission in turnips via its insect vector *C. tenellus*, is insect-transmissible. BR3-M, derived by passage in liquid medium 43 times, is also a transmissible line. The lines BR3-G (maintained in periwinkle plants by graft transmission) and BR3-P (passed in liquid medium more than 130 times) are insect non-transmissible [46]. *S. citri *R8A2, isolated from citrus in Morocco [47], and its non-helical derivative ASP-1 (both obtained from R.E. Davis, USDA/ARS, Beltsville, MD), are non-transmissible. Also provided by R.E. Davis were *S. citri *Beni Mellal, originally isolated from *C. hematoceps *collected in Morocco; *S. melliferum *TS2, isolated from honeybees and *S. floricola *23-6, isolated from a flower surface [48]. *S. phoeniceum *P40, a gift from G. Gasparich (Towson University, Towson, MD), was originally isolated from periwinkle in Syria [11]. *S. kunkelii *CR2-3X was isolated by one of us (J. Fletcher) from stunt-diseased corn collected in Costa Rica [49]. All spiroplasmas, except *S. kunkelii *CR2-3X, were grown in LD8 broth medium [50] at 31°C. The latter was grown in LD8A3 broth medium [51] at 28°C.

### Purification of chromosomal and extrachromosomal ds DNAs from spiroplasmas

For Southern blot hybridization and PCR, extrachromosomal double-stranded (ds) DNA of spiroplasma strains was isolated using the QIAprep Spin Miniprep and the QIAGEN Plasmid Mini Kits (Qiagen, Santa Clarita, CA), following the manufacturer's protocols. For primer walking, *S. citri *BR3-3X extrachromosomal DNA was isolated using a previously published procedure [28]. The isolation of chromosomal DNA from *S. citri *BR3-3X cells was performed according to Murray and Thompson [52] and using 1.4 M NaCl, 2.5% cetyltrimethylammonium bromide [CTAB], 100 mM Tris-HCl, pH 8.0, and 20 mM EDTA in the extraction buffer.

### PCR and sequencing using *S. citri *BR3-3X plasmid and chromosomal DNAs

To amplify the 3'- and flanking regions of *arp *genes from *S. citri *BR3-3X plasmid and chromosomal DNAs, two oligonucleotides were designed, forward (#7686) 5'-AACACTATTTTCACTGCGG-3', from the *S. citri *BR3-T *arp1 *sequence (GenBank accession number AJ297706), and reverse (#7960) 5'-TTTTCCATTGTTTTTGTCTCC-3', from the sequence homologous to ORF4 from the plasmid pSKU146 (pSKU146_11; accession number NC_006400). The PCR was carried out in a DNA thermal cycler (MJ Research, Waltham, MA) performing 35 cycles, each of 30 sec at 94°C, 1 min at 42°C and 3 min at 72°C. Reactions were performed separately in a volume of 50 μl containing 2.5 Units Taq polymerase (Promega), 0.20 μM primers, 200 μM of each dNTP, 1.5 mM MgCl_2_, and 100–150 ng BR3-3X plasmid and ~ 3.5 μg chromosomal DNA. The amplicons were sequenced using ~ 100 ng of each of the PCR products, 10 μM of the same primers used in the PCR in separate reactions by the ABI PRISM BigDye Terminator Cycle Sequencing method (version 1.0, Applied Biosystems, Foster City, CA) with an ABI PRISM 3700 Automated DNA Analyzer (Perkin Elmer Biosystems, Foster City, CA).

### Southern blotting

Extrachromosomal DNA of each spiroplasma strain was digested with *Eco*RI (Life Technologies, Inc.) for 4 h at 37°C. The fragments were separated by electrophoresis on a 0.75% (w/v) agarose gel in 1× TAE running buffer and transferred to Hybond-N^+ ^nylon membranes (Amersham Biosciences, Uppsala, Sweden) according to standard procedures. The blots were subsequently hybridized to Dig-11-UTP-labeled *arp1*-derived and whole-plasmid probes, labeled using a DIG DNA Labeling Kit (Roche Molecular Biochemicals, Indianapolis, IN), following the manufacturer's instructions. The *arp1*-derived probe was obtained by PCR, using clone pP89B (an *Rsa*I fragment of *S. citri *BR3-T genomic DNA; [15]) as template, and primer pair T7 and #7483 (5'-TTTAACATCAACCGAACCC-3'). The probe comprised 657 bp of a DNA segment from *S. citri *BR3-T (AJ297706; positions 2315–2989) and 72 bp derived from the cloning vector (pBluescript). PCR was carried out in a DNA thermal cycler performing 34 cycles, each of 30 sec at 94°C, 30 sec at 54°C and 1 min at 72°C. Reactions were performed in a volume of 50 μl containing 1 Unit Taq polymerase, 0.25 μM primers, 250 μM of each dNTP, 50 – 100 ng template DNA, and 2.5 mM MgCl_2_. Hybridizations were performed at 55°C in Church buffer (0.5 M sodium phosphate buffer, pH 7.2, 7% SDS, and 1 mM EDTA) overnight followed by four washes, each of 20 min, at 55°C in washing buffer (40 mM sodium phosphate buffer, pH 7.2, containing 0.1% SDS). Detection of the DIG-labeled probes was performed using a DIG Luminescent Detection Kit (Roche) following the manufacturer's protocol.

### Complete nucleotide sequencing of pBJS-O

The sequence AJ297706 was used to design primers to initiate primer walking to completely sequence and characterize the unknown portion of pBJS-O. The sequencing reactions were performed using ~ 1.2 μg of pBJS-O DNA and 40 μM of primers with the ABI PRISM BigDye Terminator Cycle Sequencing method and the ABI PRISM 3700 Automated DNA Analyzer, as mentioned above. The total 134 sequence reads with an average length of 600 bases gave us about 6× coverage of the entire plasmid sequence. The fragments were assembled from the trace files using the software package PipeOnline 2.0 [53]. Physical gaps in the sequence were closed by PCR and cloning of the products into vector pGEM-T (Promega). The clones were sequenced using primers T7 and SP6. The consensus sequence of the final assembly was annotated using the BLASTX search program [54] and the ORF Finder tool at NCBI, in which a minimum length of 100 bases was used for the nucleotide sequence of a putative ORF. The nucleotide and amino acid sequence analysis tools offered by the Biology Workbench at the San Diego Supercomputer Center, such as ClustalW and PHYLIP for generating the unrooted phylogenetic tree of the *S. citri arp *sequences, were used to further analyze the plasmid and the polypeptide sequences. BLASTN and BLASTP searches were carried out to find out relationships with the closest homologs. *S. kunkelii *CR2-3X genome sequence data were accessed and BLAST searches were performed at the Spiroplasma Genome Sequencing Project Web site mentioned above.

## Authors' contributions

BDJ performed isolation, distribution and sequence characterization of pBJS-O. JR carried out pBJS-O sequencing and assisted BDJ in primer design and sequence assembly. MB performed *S. citri *BR3-T *arp2 *gene cloning and sequencing, and also assisted BDJ in pBJS-O distribution experiments. BDJ, MB, UM and JF planned the research, BDJ and UM wrote the manuscript and MB and JF reviewed it.

## Note added in proof

During review of this manuscript sequences of plasmids from a different strain of *S. citri *were released [GenBank:AJ969069, GenBank:AJ969070, GenBank:AJ969071, GenBank:AJ969072, GenBank:AJ969073, GenBank:AJ969074].
